# Supplementary material for: Efficient and specific oligo-based depletion of rRNA
Source: Sci Rep. 2019 Aug 22;9:12281. doi: 10.1038/s41598-019-48692-2 (PMC6706579; doi:10.1038/s41598-019-48692-2)
Supplement: Supplementary file 1 — Supplementary Information [file 41598_2019_48692_MOESM1_ESM.pdf]

Efficient and specific oligo-based depletion of rRNA

Amelie J. Kraus, Benedikt G. Brink and T. Nicolai Siegel

Supplementary Information

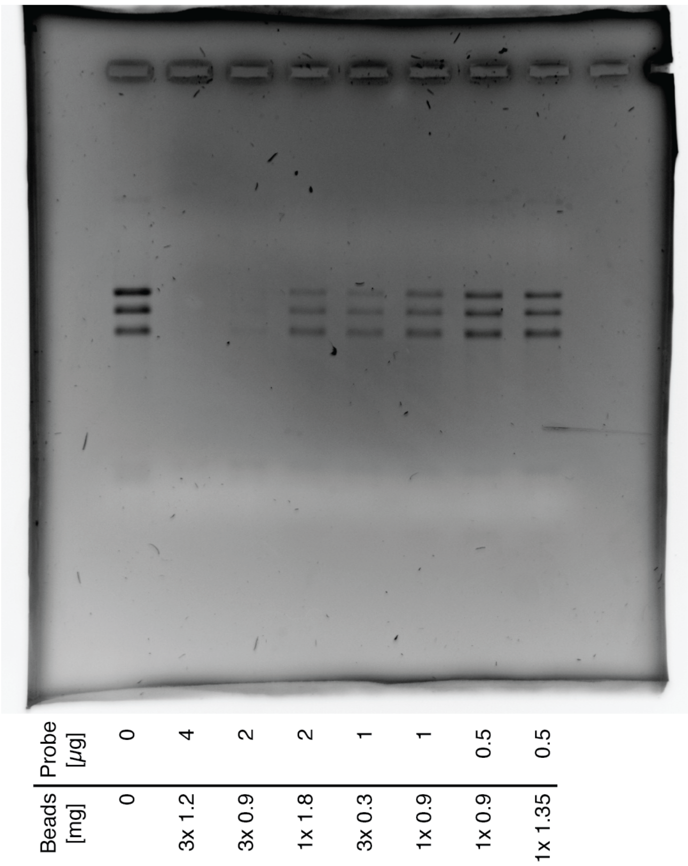

Supplementary Figure 1.

Uncropped agarose gel revealing presence or absence of the three large trypanosomal rRNA transcripts following different depletion conditions. For each condition 2 µg of total RNA was used.

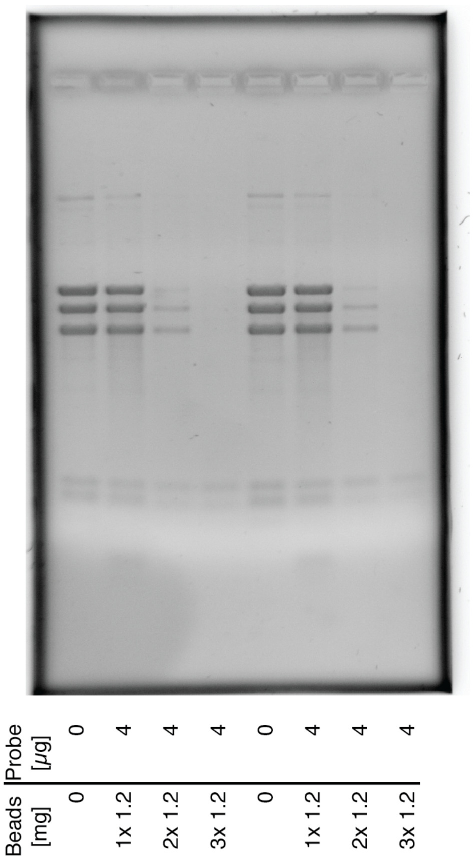

Supplementary Figure 2.

Uncropped agarose gel revealing presence or absence of the three large trypanosomal rRNA transcripts following different depletion conditions. For each condition 2 µg of total RNA was used. All eight samples shown in this figure were analyzed by RNA-seq.

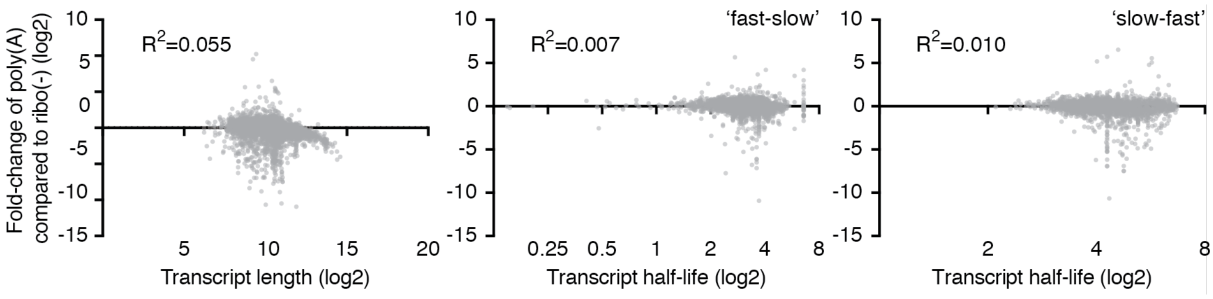

Supplementary Figure 3 Correlation analysis of poly(A)-enriched/ribo(-) transcript levels vs transcript length or transcript half-life. The difference in transcript levels (fold-change) between the Hutchinson et al. [1] measurements and our measurements was plotted vs transcript length (left panel) and half-life of genes classified as following a degradation pattern of 'fast-slow' (middle panel), or genes following a 'slow-fast' pattern. Half-life measurements are based on Fadda et al. [2].

References:

1. Hutchinson, S., Glover, L. & Horn, D. High-resolution analysis of multi-copy variant surface glycoprotein gene expression sites in African trypanosomes. *BMC Genomics* **17**, 806 (2016).
2. Fadda, A. et al. Transcriptome-wide analysis of trypanosome mRNA decay reveals complex degradation kinetics and suggests a role for co-transcriptional degradation in determining mRNA levels. *Mol Microbiol* **94**, 307-326 (2014).
